# Retrospective Vibration Vector Analysis of Periodic Synchronous Vibration in Nuclear Steam Turbine Generator Units

**DOI:** 10.3390/s26216856

**Published:** 2026-10-29

**Authors:** Hua Lin, Jiang Guo, Wei Wang

**Affiliations:** 1 School of Power and Mechanical Engineering, Wuhan University, Wuhan 430072, China; linhua@snpas.com.cn; 2 State Nuclear Power Automation System Engineering Co., Ltd., Shanghai 200241, China; wangwei@snpas.com.cn

**Keywords:** nuclear steam turbine generator, rotor dynamics, periodic unstable vibration, synchronous vibration, Morton effect, seal-ring thermal effect, thermal bow, vibration vector decomposition, Powell optimization, condition monitoring

## Abstract

This retrospective study reanalyzes 59 archived synchronous (1X) amplitude–phase observations from two half-speed nuclear steam turbine generator units: 25 Type-A 6H and 34 Type-B 8H points. A constant complex response and a rotating slow component are fitted jointly to the reconstructed real and imaginary parts using Powell optimization. The baseline magnitudes are 27.96 and 82.04 μm, the slow-component magnitudes are 8.11 and 6.21 μm, and the periods are 340.27 and 365.02 min, respectively. Full-record amplitude R^2^ values are 0.918 and 0.883. With the last approximately 30% of each record withheld, the amplitude RMSE values are 4.19 and 2.85 μm and the R^2^ values are −0.782 and 0.185, with modest improvement over a simple periodic amplitude fit. Residual structure and time-window sensitivity limit prediction. Archived seal inspections and cooling trends provide context for competing physical explanations. Neither the decomposition nor the conditional film-thickness illustration identifies a unique heat source or validates a seal-to-bow causal chain. The results support description and comparison of the retained response patterns.

## 1. Introduction

Nuclear steam turbine generator units are key energy-conversion equipment in the secondary loop of nuclear power plants, and the operating stability of the shafting is directly related to unit safety, economic performance, and planned maintenance. The investigated ARABELLE-type unit belongs to a half-speed nuclear steam-turbine platform with a combined high- and intermediate-pressure module and multiple low-pressure modules [1]. Its long, multi-support shafting motivates examination of the interaction among local excitation, rotor response, and support conditions. Investigating slowly varying synchronous vibration in this type of unit has practical significance for operation and maintenance.

Existing studies have analyzed steam-flow excitation in turbine units [2], foundation vibration isolation [3], valve-group instability [4], vibration-probe bracket resonance [5], rotor–stator rubbing and thermally induced vibration [6,7,8], low-pressure-cylinder thermal deformation [9], and balancing treatment [10]. These mechanisms require different diagnostic evidence even when their measured responses contain a strong 1X component. Vibration evaluation also depends on the measurement quantity and operating state. ISO 20816-2 addresses broad-band shaft and bearing-housing vibration for large turbine generator sets [11]; its criteria cannot be applied directly to a decomposed 1X component without matching the channel, amplitude convention, and plant protection settings. The monitoring problem examined here is the interpretation of slowly changing 1X amplitude and phase in sparse trend records.

For thermally induced rotor vibration, studies of generator seal rings and bearing oil films have linked circumferential journal heating to the rotor thermal bow [12,13,14,15,16]. International work provides more direct evidence of this feedback. Tong and Palazzolo measured circumferential journal temperatures with 20 sensors in a tilting-pad bearing test rig and identified a speed-dependent phase separation between the temperature maximum and the location experiencing the smallest time-averaged film thickness [17]. Subsequent studies showed that pivot flexibility and friction affect the predicted instability range [18], while flexure-pivot models coupled bearing deformation to the thermal bow of the complete rotor and were benchmarked against experiments [19]. Yang and Palazzolo also demonstrated the importance of pad-inlet temperature distributions and oil-injection arrangements for Morton-effect predictions [20].

Thermal bending can produce different vibration trajectories. Controlled induction-heating experiments reproduced stable, unstable, and limit-cycle spiral responses; their periods depended on heating magnitude and phase, modal damping, and proximity to a critical speed [21]. A slowly changing 1X vector is therefore compatible with thermal feedback but does not identify its heat source. Labyrinth-seal studies emphasize flow-induced cross-coupled forces and their sensitivity to surrounding flow [22], whereas recent simulations of active magnetic bearings couple electromagnetic losses to rotor heating and bending [23]. These mechanisms differ from viscous heating in an oil seal. Their results cannot be transferred quantitatively to a 1500 r/min, multi-support nuclear turbine generator without matching the rotor, bearing, and seal conditions.

Recent work also addresses generator-specific oil films. Li et al. coupled shear-flow heating, thermal bending, and rotor response with an oil-temperature adjustment test on a nuclear generator [24]. Zhang et al. found that eccentric clearance, temperature-dependent viscosity, and fluid–solid heat exchange govern non-uniform heating and floating behavior in a double-flow hydrogen ring seal [25]. The latter configuration differs from the single-flow seal considered here, but it identifies relevant coupling mechanisms and parameters.

This paper retrospectively examines periodic synchronous vibration in two ARABELLE-type unit records. A linear response framework provides physical context, while a complex-vector decomposition is applied directly to the 59 retained 1X amplitude and phase observations. The contribution is a reproducible signal-analysis procedure that distinguishes a constant response baseline from a slow component, checks amplitude and phase consistency, and evaluates residuals, temporal hold-out performance, and sensitivity to the precision of the reported measurements. Archived seal inspections, cooling-adjustment trends and a separate balancing record contextualize competing explanations. The response fit does not depend on the archived finite-element calculation or identify a unique excitation source.

## 2. Materials and Methods

### 2.1. Investigated Unit, Seal-Ring Configuration, and Field Data

During normal generator operation, the internal hydrogen pressure is approximately 0.3 MPa(a), and the seal-oil pressure is approximately 0.35 MPa(a). The shaft-sealing devices at both ends of the generator employ single-flow annular seal rings, which mainly consist of the air-side seal ring, hydrogen-side seal ring, and seal-ring chamber. Both the air-side and hydrogen-side seal rings consist of four 90° arc segments and form an approximately circular structure under the constraints of the backing springs and seal-ring chamber, while maintaining a small radial clearance from the generator rotor journal. Seal oil enters from the middle of the air-side and hydrogen-side seal rings, flows axially to both sides, and then returns to the main oil tank together with the lubricating oil. Under normal operating conditions, the seal ring can float slightly with the journal position under hydrodynamic lubrication, thereby maintaining relatively stable oil-film thickness and pressure distribution between the seal ring and the journal.

Field 1X amplitude and phase records from Type-A and Type-B units are analyzed as two available historical cases. They were included because their complete elapsed-time, amplitude and phase tabulations survived. The archive describes full-power operation at the rated speed of 1500 r/min with relatively stable process conditions; these are reported historical conditions, not a prospectively audited selection protocol. The original sampling frame and inclusion/exclusion log cannot be reconstructed, so case-representativeness and selection bias cannot be evaluated. The retained Type-A and Type-B records span 0–693 and 0–1008 min, respectively. The records are unevenly spaced: Type-A intervals range from 10 to 65 min, with a median of 26.5 min; Type-B intervals range from 15 to 78 min, with a median of 28 min. These are trend-record intervals, not the sampling rate of the underlying vibration waveform. The tabulated amplitude and integer-degree phase values are retained, with no smoothing, interpolation, or outlier removal before fitting. Each record has its own elapsed-time origin, and no simultaneous acquisition between units is assumed.

The measurement system uses eddy-current probes for shaft-relative displacement, with a nominal sensitivity of 8 mV/μm. Each of the eight bearings has nominally orthogonal H and V probes, oriented approximately 5° from the horizontal and vertical reference directions. Shaft-vibration amplitudes are reported as peak-to-peak displacement. Bearing-housing vibration is measured separately by piezoelectric acceleration sensors with a nominal sensitivity of 100 mV/g and is converted to RMS velocity. The present decomposition uses the exported shaft 1X amplitude and Keyphasor-referenced phase; bearing-housing RMS values are not inputs to the phasor fit.

Type A uses the 6H channel and Type B uses 8H, as explicitly labeled in the archived monitoring workbook. All 25 Type-A and 34 Type-B elapsed-time, amplitude and degree-phase records are retained unchanged and listed in the corresponding data tables in Section 3. The Type-B assignment is also consistent with the archived monitoring-plot label and all 34 amplitude–phase pairs after one common elapsed-time translation. This comparison applies no amplitude rescaling, phase offset, or time-axis stretching. The first observation defines the time origin of each record. A phase reference relates the synchronous response to a once-per-revolution signal [26]; a common mechanical phase-zero and lead/lag convention between the two units is not established. Fitted angles retain each record’s convention and are not balancing correction angles.

Measurement uncertainty cannot be inferred from fitting residuals or from printed resolution alone. The GUM framework propagates uncertainties assigned to measurement inputs, which may be evaluated by statistical (Type A) or other (Type B) methods [27,28]. Nominal probe sensitivity does not establish calibration uncertainty. Equal observation weights are used because channel-specific calibration uncertainties are not defined for these records. Section 2.5 evaluates the contribution of the reported amplitude and phase precision under an explicit rounding assumption, separately from the residual-based intervals and the sensitivity to record selection.

### 2.2. Linear Response Framework and Archived Context

The operating speed of 1500 r/min corresponds to a synchronous frequency of 25 Hz. Equations (1)–(4) provide an idealized linear description of how a prescribed bow can contribute to synchronous displacement. They introduce the mechanical superposition argument used below; the retrospective analysis itself is performed directly on the recorded 1X phasors. With small linear displacements, disks, elastic shaft segments and rigid connections are represented by fixed matrices. Gyroscopic terms are retained, while explicit dissipative damping and coupled heat generation are omitted. Fast mechanical time τ is measured in seconds. The displacement equation is written as Equation (1).

(1) $$M\ddot{x}+G(\Omega )\dot{x}+Kx=K_{r}x_{b}(\tau )$$

Here, *M* is the assembled mass matrix, *G*(*Ω*) is the speed-dependent gyroscopic matrix, *K* is the assembled linear stiffness matrix, Kᵣ is the rotor-body stiffness matrix, *x* is the generalized displacement vector, *xᵦ* is the prescribed thermal-bow vector, and *Ω* is the shaft angular speed in rad/s. The right-hand side represents an equivalent mechanical excitation due to the prescribed bow; it does not calculate the temperature field that creates the bow.

Writing *x*(*τ*) and *xᵦ*(*τ*) as the real parts of harmonic complex amplitudes multiplied by exp(*iΩτ*), where *i*^2^ = −1, gives the frequency-domain equation in Equation (2).

(2) $$[K-\Omega^{2}M+i\Omega G(\Omega )]\hat{x}=K_{r}\hat{x_{b}}$$

Define the dynamic-stiffness matrix *D*(*Ω*) and its inverse, where nonsingular, as in Equation (3), obtained directly from Equation (2).

(3) $$D(\Omega )=K-\Omega^{2}M+i\Omega G(\Omega ),\hat{x}={D(\Omega )}^{-1}K_{r}\hat{x_{b}}$$

The displacement response in a specified measurement direction j is then obtained using the displacement-selection vector *c_j_*, as shown in Equation (4).

(4) $$\hat{s_{j}}={c_{j}}^{T}\hat{x}={c_{j}}^{T}{D(\Omega )}^{-1}K_{r}\hat{x_{b}}$$

Equation (4) expresses a linear transfer from a prescribed bow to displacement in a probe direction. The transfer depends on the bow shape, support properties, operating speed and measurement direction, so a common excitation can produce different responses at different stations. This framework motivates response-vector superposition. It does not supply a thermal evolution law or a unit-specific numerical transfer function for the present records.

**Figure 1:**
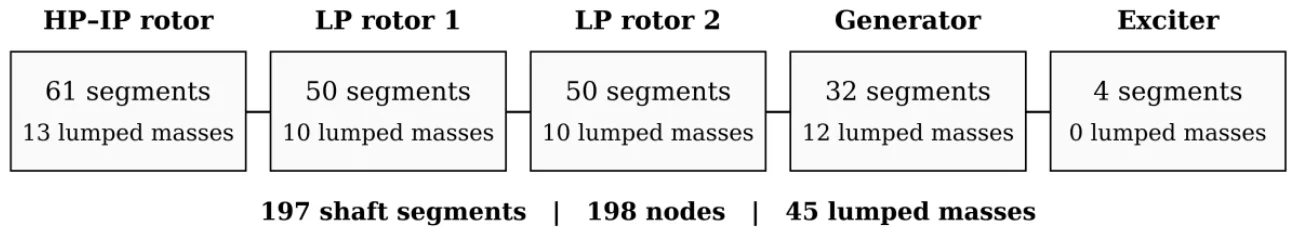
Archived shafting discretization summary. Numbers denote reported shaft segments and added masses. The schematic is not to scale.

The archived shafting description reports the nominal rotor properties and discretization summarized in Table 1 and Figure 1. It contains 197 shaft segments, 198 shaft nodes and 45 added masses, with eight further nodes representing bearing–foundation connections. These reported characteristics describe the investigated machine and its historical modeling context. The trend-analysis model does not use the archived finite-element matrices or simulated response amplitudes.

HIP denotes the combined high- and intermediate-pressure rotor; LP1 and LP2 denote the two low-pressure rotors. The listed lengths are nominal rotor-level quantities. Exciter length and material properties are not specified in the source table.

In a beam representation, distributed shaft properties, lumped masses and support connections determine the response operator. The displacement-selection vector in Equation (4) extracts the translation in the probe direction. As distinguished in the DyRoBeS documentation [29], model parameters and output display settings are separate parts of a numerical calculation. Here, the surviving model descriptions and plots are retained as archived context.

Appendix A collects the archived support-bearing and foundation data: bearing coefficients, reported oil-temperature rises, critical-speed summaries, design dimensions and isolator properties. The three-pad support-bearing coefficients were reported from a finite-difference calculation coupling film thickness, Reynolds pressure, energy, and pad moment balance at 1500 r/min and 45 °C. They concern the support bearings, not the generator seal rings. Their coefficient ratios and tabulated stiffness sensitivity are evaluated descriptively; none enters the phasor-fitting objective.

**Figure 2:**
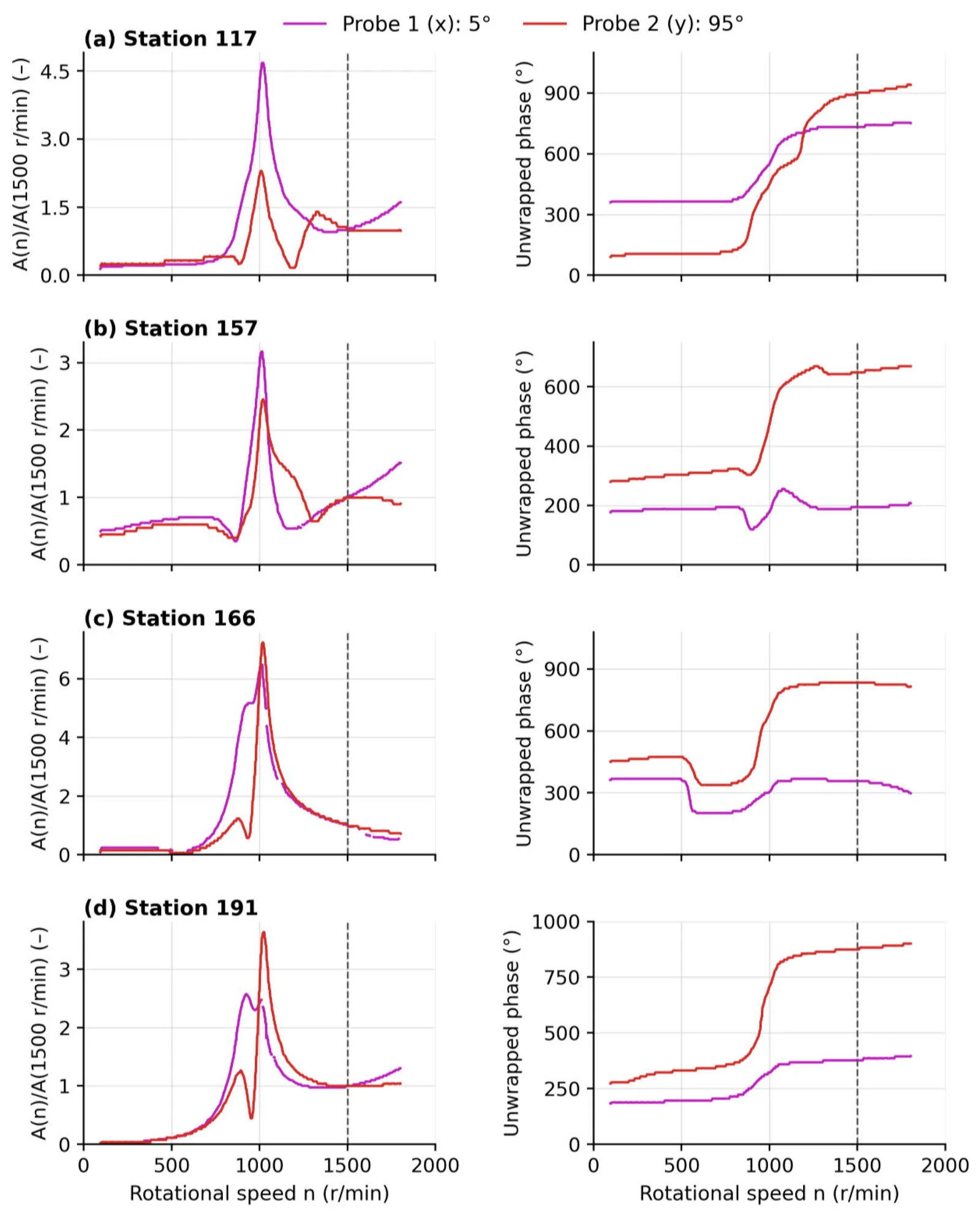
Digitized archived synchronous-response curves at stations (**a**) 117, bearing No. 5; (**b**) 157, No. 6; (**c**) 166, No. 7; and (**d**) 191, No. 8. Left: Each probe amplitude divided by its own value at 1500 r/min. Right: The archived unwrapped phase in degrees. Dashed vertical lines mark 1500 r/min. Probe 1 and Probe 2 follow the archived directions x, 5°, and y, 95°, respectively. Gaps preserve overlapping or obscured source traces. The curves provide historical response-shape context and are not fitted to the field records.

Figure 2 preserves the shapes of the archived speed-response plots using A(n)/A(1500 r/min), where n is shaft speed. The ordinate is dimensionless, and phase retains the source convention. A ±4-pixel ordinate perturbation changes the digitized reference amplitudes by approximately 2.8–9.3%, depending on the probe. This normalization retains within-curve speed dependence while removing each curve’s constant amplitude scale. Appendix B reports a separate common-reference summary of relative amplitudes under its stated display assumptions. Neither representation is used to calibrate the measured 1X response or select the fitted slow period.

Linear scaling follows directly from Equation (4). For a fixed bow shape, input location, support state and mechanical frequency, multiplying the prescribed bow by a positive constant α multiplies its incremental displacement response by α and leaves its phase unchanged wherever the response is nonzero. A common positive input scaling therefore cancels in a normalized response ratio. This algebraic property explains the role of normalization without assigning a physical bow amplitude to either field record.

### 2.3. Physical Background and Conditional Film-Thickness Illustration

Seal-ring thermal feedback is considered here as a literature-supported explanation, not an identified cause of the two archived records. Existing studies relate eccentric clearance to local film thinning, viscous heating and circumferential journal-temperature differences [12,24,25]. The resulting thermal forcing depends on geometry, lubricant supply, heat removal and the motion of both journal and ring. These dependencies provide physical context for the response analysis without specifying the operating state of the investigated seals.

The relevant thermal forcing is the circumferential component that persists in rotor coordinates during synchronous whirl [16,17]. Eccentric clearance changes both viscous dissipation and convective heat removal, so the resulting temperature field depends on the oil viscosity, clearance, pressure-driven flow, and heat exchange with the solids [25]. A temperature difference then produces differential thermal expansion and bending; an equivalent bow or thermal moment connects this heat-transfer problem to the mechanical response. This sequence explains why oil-film heating is a plausible source of slowly changing 1X response while preserving the distinction between stationary seal hot regions and rotor-fixed thermal asymmetry.

Axial skew, ellipticity, installation clearance, and friction at the seal-ring support surfaces can alter the oil-film pressure distribution and restrict floating [13]. A biased oil film may then concentrate viscous heating. However, a stationary thin-film region does not by itself establish a persistent temperature difference fixed to the rotating journal. In the Morton mechanism, synchronous whirl can repeatedly expose the same shaft surface region to stronger heating, producing a circumferential temperature difference in rotor coordinates [16]. Restricted seal-ring motion is a possible boundary condition in this process. The inspection evidence described below is consistent with impaired mobility, but the operating floating displacement was not measured. Direct rotor–stator rubbing introduces a separate frictional heat source and is not automatically classified as the Morton effect.

The unit-specific inspection records describe oil residue, polished contact patches, local grooves, and solidified adhesive around the seal-ring contact surfaces. The maintenance specification gives nominal radial clearances of 0.08 mm on the air side and 0.15 mm on the hydrogen side, with a polished surface roughness requirement of Ra ≤ 0.8 μm. These are assembly specifications, not measured running oil-film thicknesses. A separate temperature survey reports support-bearing pad temperatures of 71–85 °C at bearings No. 1–6, 94/102 °C at the two monitored ends of No. 7, and 98/101 °C at No. 8. These pad-metal values describe the support-bearing thermal state and must not be interpreted as seal-ring or circumferential journal temperatures. The inspections and cooling observations in Section 3.5 make generator-end thermal sensitivity plausible, but do not distinguish viscous heating in the seal from rubbing-induced heating, support-bearing thermal effects, or changes in mechanical transmission.

A conditional film-thickness estimate uses the smooth, parallel-axis thin-gap geometry [24], with eccentricity ratio *ε = e/c* and minimum gap *h_min_ = c(*1 *− ε)*. Here, e is the journal-to-ring center offset and c is the nominal radial clearance. For the air/hydrogen-side clearances of 80/150 μm, assumed ε = 0.5 gives minimum gaps of 40/75 μm, and ε = 0.8 gives 16/30 μm. With viscosity μ and relative surface speed v_s_ fixed, laminar Couette dissipation per unit surface area is *p_C_ = (μv_s_*^2^*)/(h)* (W/m^2^); its value at the minimum gap is, respectively, two and five times the concentric-film value. These are assumed geometry scenarios, not measured operating eccentricities. They omit pressure-driven flow, property changes, deformation and ring motion, and do not determine the temperature field or heat absorbed by the rotor.

A slowly changing bow can alter the synchronous response at a measuring point. Under a fixed linear transfer relationship, this contribution can be superposed with a stable baseline response. The geometric argument below explains how changing the relative phase may either increase or reduce the resultant amplitude, without identifying a unique excitation source.

Let *U* denote a stable complex displacement-response vector, *V*(*t*) a slowly varying response vector, and *Y*(*t*) their resultant in the same phase-reference convention. All three quantities are displacement responses in micrometers, rather than unbalance masses or force vectors. Their superposition is given in Equation (5).

(5) Y(t)=U+V(t)

At the initial observation time *t*_0_, the resultant vector is given by Equation (6).

(6) $$Y_{0}=U+V_{0}$$

In the separate spring-foundation calculation, the generator critical speeds are 955 and 2555 r/min (Table A3), with 1500 r/min between them. This fact alone does not determine the phase lag at every measuring point in a multi-mode shafting system. The lag depends on the transfer response, modal participation, and support properties. If the slow response changes from *V*_0_ to *V*_1_ while *U* remains fixed, the resultant at the next observation time follows Equation (7).

(7) $$Y_{1}=U+V_{1}=Y_{0}+(V_{1}-V_{0})$$

**Figure 3:**
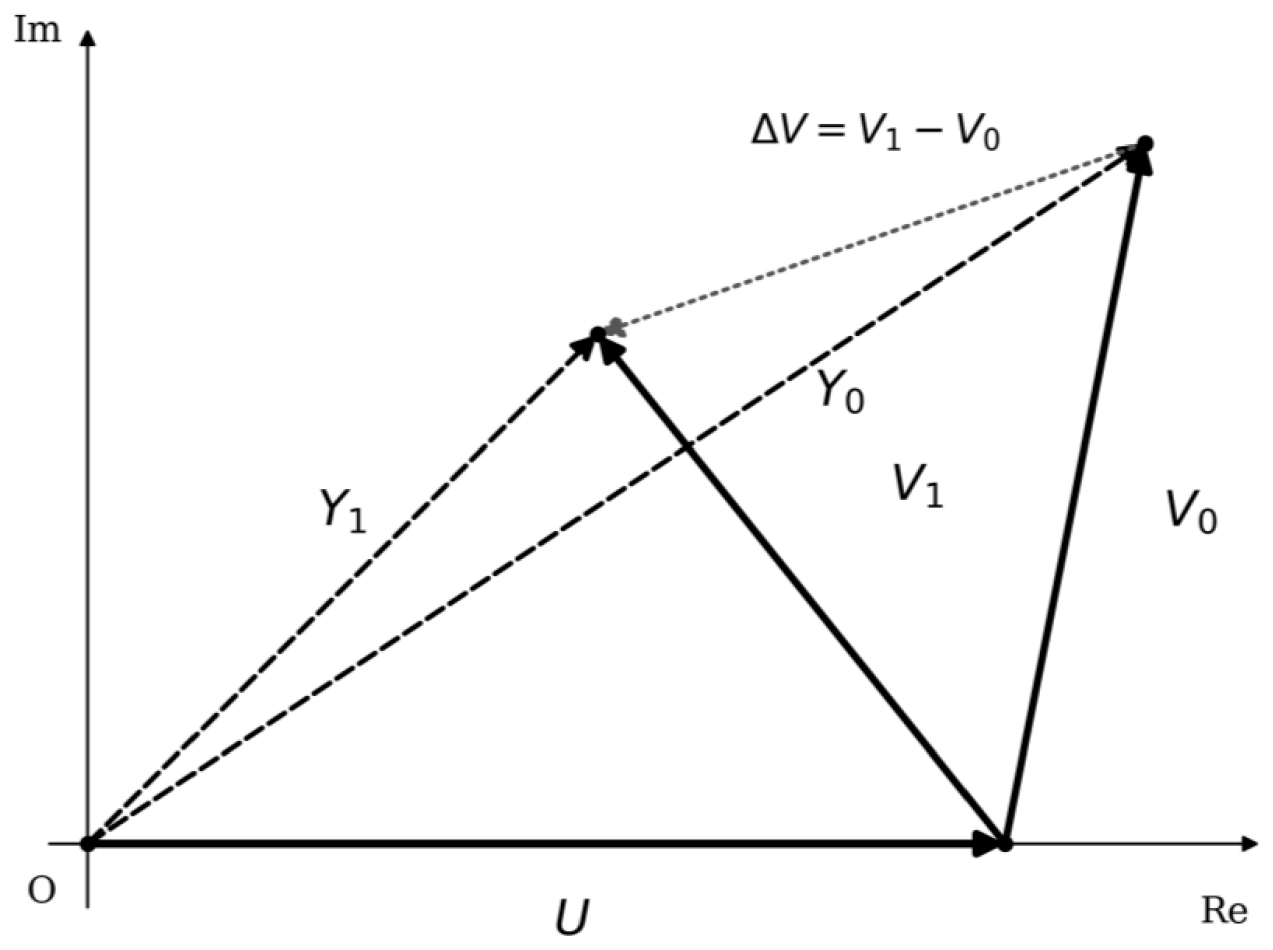
Response-vector superposition at two observation times. *U* is the stable baseline response; *V*0 and *V*1 are the slowly varying response vectors at the initial and subsequent observation times, respectively; *Y*_0_ and *Y*_1_ are the corresponding resultant response vectors; the change is *ΔV* = *V*_1_ − *V*_0_. Arrows are illustrative phasors, not measured forces or a shaft orbit.

The resultant may increase or decrease depending on the relative response phases. Figure 3 illustrates this vector relationship at two observation times; it does not prescribe a thermal-feedback evolution law or demonstrate a stability boundary.

### 2.4. Vibration-Vector Decomposition and Powell Optimization

The recorded 1X amplitude *A_i_* and phase *φ_i_* are reconstructed as complex response phasors *z_i_* = *A_i_* exp(*iφ_i_*) using the integer-degree phases converted to radians. The real and imaginary parts are quadrature coordinates of one measured 1X channel, not two independently measured radial shaft directions. Both coordinates are fitted simultaneously so that the reported amplitude and phase are used consistently.

The fitted response consists of a constant complex baseline *C* and a slow term *D* exp(*iωt*) of constant magnitude, with elapsed time t in minutes. In this first-order approximation, *C* = *P*_1_ exp(*iθ*) and *D* = *P*_2_ exp(*iβ*), with *P*_1_ and *P*_2_ nonnegative. These are displacement-response magnitudes, not mass-unbalance values. The frequency *ω* = ±2*π*/*T* describes the slow trend and is distinct from the mechanical shaft speed *Ω*. The recorded phase origin is retained; no correspondence with the minimum-clearance direction is assumed.

For *t′ = t − Δt* and a constant phase/gain conversion *z′(t′) = g e^iψ^ z(t′ + Δt)* with *g* > 0, Equation (8) retains its form with *C′ = g e^iψ^ C* and *D′ = g e^i(ψ + ωΔt)^ D*. Thus, the fitted period *T*, the ratio *P*_2_/*P*_1_, and amplitude R^2^ are unchanged. Response magnitudes and absolute residuals scale with g. A phase-sign reversal instead conjugates the coefficients and reverses the fitted sign of *ω*, both of which are permitted in the search. These properties require a constant conversion within the record; gain drift, time-dependent phase error, and clock-rate error do not cancel.

**Figure 4:**
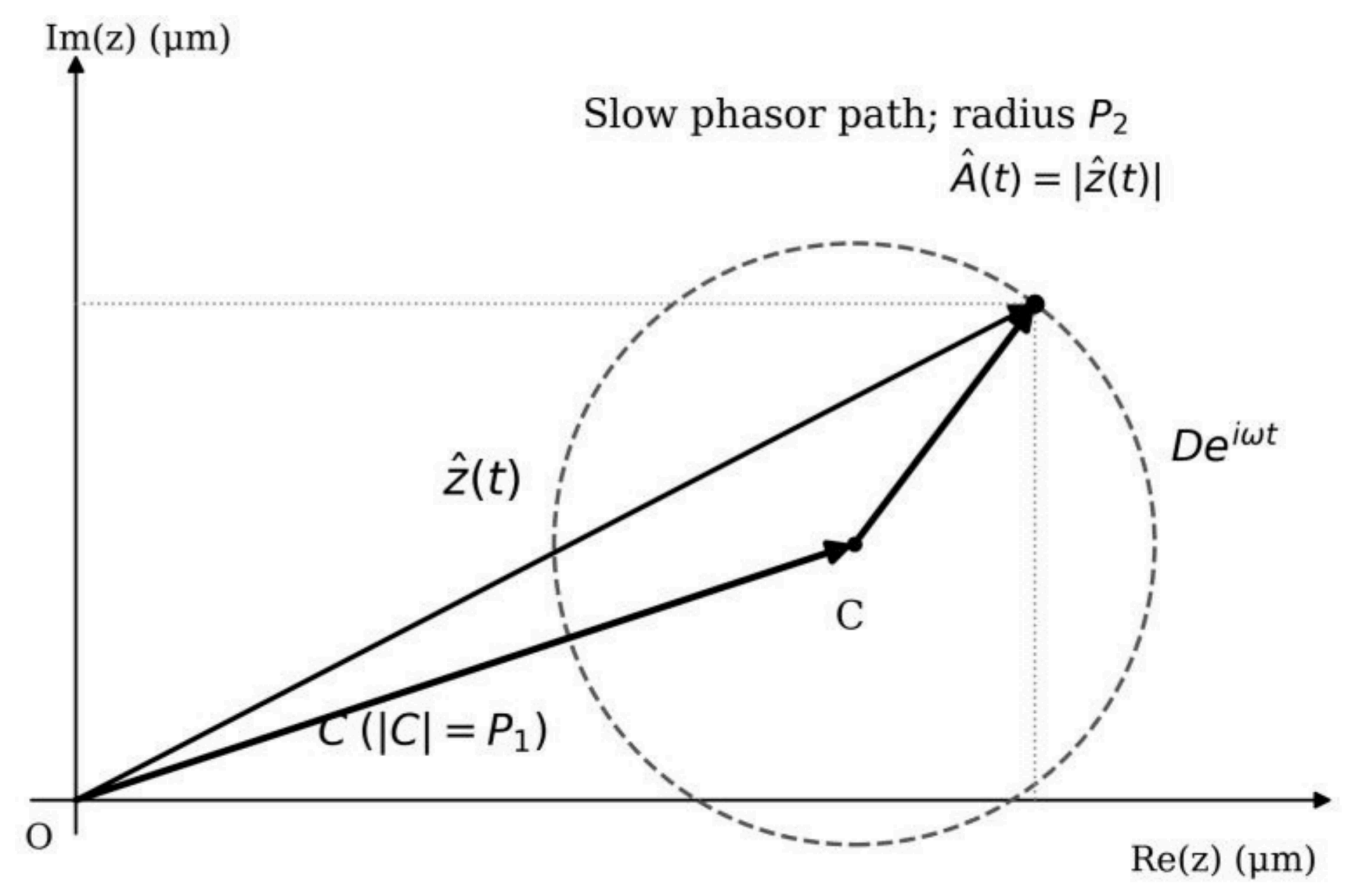
Complex response model and measured phasor coordinates. The solid arrows represent the measured complex phasors $\hat{z}(t)$ and the constant baseline vector *C*. The dashed circle represents the idealized slow path of $\hat{z}(t)$ about *C* with radius *P*_2_. *P*_1_ is the magnitude of *C*; the measured 1X amplitude is the distance from the origin to z_i_.

The complex response model is written as Equation (8) and illustrated in Figure 4. The five real fitting parameters are *Re*(*C*), *Im*(*C*), *Re*(*D*), *Im*(*D*), and the slow period *T*, with the two rotation senses evaluated separately.

(8) $$\hat{z}(t)=C+De^{i\omega t}=P_{1}e^{i\theta }+P_{2}e^{i(\omega t+\beta )}$$

The nonnegative model amplitude $\hat{A}(t)$ is the modulus of Equation (8), as shown in Equation (9). The signed imaginary projection is *Im*[$\hat{z}(t)$] = *P*_1_ *sin θ* + *P*_2_ *sin*(*ωt* + *β*). That projection is retained as a derived coordinate in the data tables of Section 3, while measured and calculated amplitudes are compared in the corresponding amplitude tables. Amplitude-only fitting would identify the phase difference *β* − *θ*, but not the two phase angles separately; joint complex fitting avoids that ambiguity.

(9) $$\hat{A}(t)=|\hat{z}(t)|=\sqrt{{P_{1}}^{2}+{P_{2}}^{2}+2P_{1}P_{2}cos(\omega t+\beta -\theta )}$$

Powell’s derivative-free method [30] is used to refine the slow period after solving *C* and *D* by complex linear least squares at each trial period. The objective is *S* = *Σ_i_* |*z_i_* − $\hat{z}$(*t_i_*)|^2^, which equals the sum of the squared real- and imaginary-coordinate residuals. Both signs of *ω* are searched over a common period range of 120–1000 min using a 0.5 min grid, followed by bounded Powell refinement around the best grid candidate. SciPy 1.17.0 (SciPy Developers, Austin, TX, USA) is used with xtol = 10^−11^, ftol = 10^−12^, maxiter = 2000, and maxfev = 20,000. The fixed range covers a broad interval of slow-trend periods; it is a modeling restriction, not a physical stability limit. The positive recorded-phase direction gives the lower objective for both cases.

For amplitude assessment, the residual is *e_i_* = $\hat{A}(t)$− A_i_, MSE = *Σ_i_e_i_*^2^/*n*, RMSE = √MSE, and R^2^ = 1 − *Σ_i_e_i_*^2^/*Σ_i_*(*A_i_* − *Ā*)^2^. Pearson r and max_i_|100*e_i_*/*A_i_*| are reported separately. Complex RMSE is √(*S*/*n*), and phase RMSE uses angle differences wrapped within −180° to 180°. Thus, the optimization objective is explicitly distinguished from the amplitude diagnostics; R^2^ is not calculated as r^2^.

Temporal assessment reserves the last n − floor(0.7n) observations without shuffling. All parameter estimation and rotation-sense selection for this test use only the earlier observations. A simpler amplitude baseline, $\hat{A}s(t)$= *a* + *b cos*(2*πt*/*T_s_*) + *c sin*(2*πt*/*T_s_*), is fitted with the same period bounds and time split. After scoring the withheld observations, the complete record is refitted solely for the descriptive parameter tables and curves. This is a retrospective within-record hold-out test, not validation on a new unit.

Sensitivity is examined using the first and last ceil(0.8n) observations, odd- and even-numbered records at their original timestamps, and ten initial periods from 150 to 950 min. Conditional 95% percentile intervals are obtained from 1000 circular moving-block resamples of centered complex residuals, using blocks of four successive observations and retaining the paired real and imaginary errors. Observation times and the selected rotation sense are held fixed, and the period is refitted over the stated bounds in every replicate; random seeds are 4617027 and 4617028. Because the records span few cycles and have irregular spacing, these intervals do not establish coverage for time-window drift, instrument error, or a causal thermal mechanism.

### 2.5. Sensitivity to Reported Amplitude and Phase Precision

The trend-data tables report amplitudes in integer micrometers and phases in integer degrees. A conditional nearest-integer rounding scenario assigns independent uniform perturbations within ±0.5 μm and ±0.5°, respectively, to every amplitude–phase pair. This assumption concerns the reported values and is not a specification of the instrument accuracy. The corresponding standard uncertainties are *u*(*A*) = 0.5/√3 μm and *u*(*φ*) = (0.5*π*/180)/√3 rad [27]. For the Cartesian coordinates *x* = *A cos φ* and *y* = *A sin φ*, the first-order covariance propagation is given by Equation (10), consistent with the multivariate GUM formulation [28].

(10) $$\Sigma_{xy}=J\left[\begin{array}{cc} u^{2}(A) & 0\\ 0 & u^{2}(\varphi ) \end{array}\right]J^{T},\;J=\left[\begin{array}{cc} cos\;\varphi & -A\;sin\;\varphi \\ sin\;\varphi & A\;cos\;\varphi \end{array}\right]$$

The square root of the covariance trace combines the two quadrature contributions; it is not the uncertainty of amplitude alone. Real and imaginary errors can be correlated even under independent amplitude and phase perturbations. To propagate the same rounding scenario into the fitted parameters, 2000 Monte Carlo refits are performed per case with random seeds 20261020 and 20261021. The original timestamps, record membership, equal weights, and positive recorded-phase rotation sense are held fixed. Each draw uses the common 120–1000 min period range, a 0.5 min grid, and bounded scalar refinement after solving the complex linear coefficients. The 2.5th and 97.5th percentiles define conditional sensitivity intervals. They are reported separately from the residual bootstrap and do not include calibration bias, waveform extraction, timestamp error, or model discrepancy.

## 3. Results

### 3.1. Analysis of Type-A Unit Data

The Type-A record is reported for the 6H measuring point, with its first observation at 20:49:55 on 25 January 2018, defined as *t* = 0. The 25 unevenly spaced observations are listed in Table 2. The first 17 observations, from 0 to 458 min, are used for the temporal training fit; the final eight, from 484 to 693 min, are withheld for assessment.

Joint fitting of the reconstructed complex phasors gives an amplitude MSE of 2.979 μm^2^, RMSE of 1.726 μm, Pearson r of 0.961, and R^2^ of 0.918. The complex RMSE is 3.438 μm, and the phase RMSE is 6.53°. These values describe the full-record fit, not independent verification. The parameter estimates and conditional intervals are listed in Table 3.

The amplitudes calculated from the refitted model are compared with the unchanged measured amplitudes in Table 4. The maximum absolute relative deviation is 18.15%, occurring at the low-amplitude observation *t* = 390 min. No general acceptability threshold is imposed: relative errors are larger when the measured amplitude is small, and the residual pattern must also be considered.

Substitution of the fitted complex coefficients gives Equation (11), with t in minutes and angular arguments in radians. Equation (12) is the equivalent amplitude expression in degrees; 360 *t*/*T* is used for a degree argument, while 2*πt*/*T* is used for a radian argument. Displayed coefficients are rounded after fitting.

(11) $$\hat{z_{A}}(t)=(1.810681-27.901503i)+(7.057242+3.991450i)e^{i0.01846513946t},\hat{A_{A}}(t)=|\hat{z_{A}}(t)|$$

(12) $$\hat{A_{A}}(t)=\sqrt{27.960194^{2}+8.107795^{2}+2(27.960194)(8.107795)cos[(\frac{360t}{340.272833}+115.778695){}^{\circ}]}$$

The Type-A baseline magnitude is 27.96 μm, and the rotating-component magnitude is 8.11 μm, with a period of 340.27 min. At *t* = 0, Equation (11) gives $\hat{z}$ = 8.867923 − 23.910054*i* μm and $\hat{A}$ = 25.502 μm, matching the first calculated value in Table 4. The imaginary projection is negative at that time and is not an amplitude. The fitted baseline phase describes the recorded 1X reference only; neither a physical heavy-spot angle nor a balancing mass can be inferred without a calibrated transfer relation.

**Figure 5:**
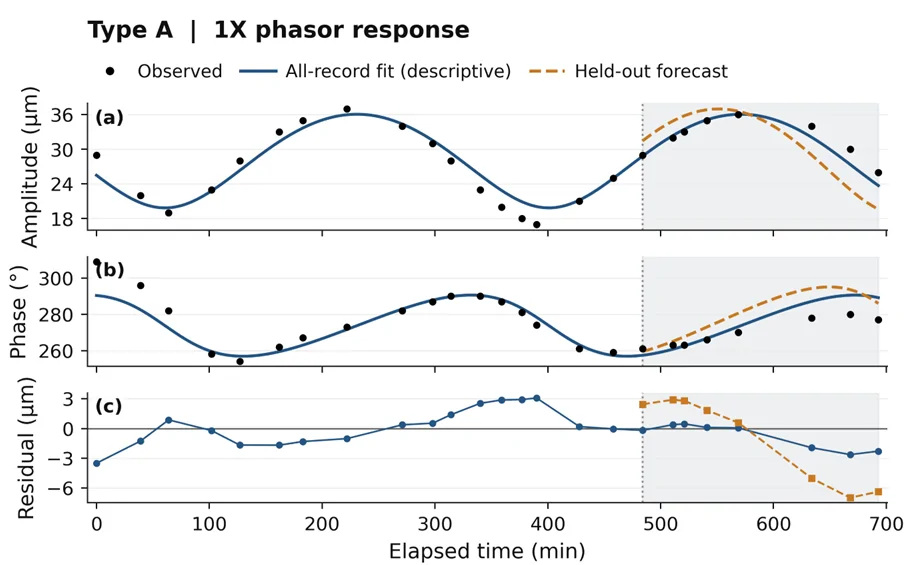
Type-A 6H amplitude, phase, and amplitude residuals. (**a**) Measured 1X amplitude versus elapsed time. Black markers are observations, blue curves are descriptive full-record fits, and orange dashed curves in the shaded interval are forecasts from the first 17 observations only; (**b**) Measured 1X phase versus elapsed time. The phase uses the recorded convention; (**c**) Amplitude residuals, calculated as predicted minus observed amplitude. Blue markers denote residuals of the full-record fit, and orange markers denote residuals of the held-out forecast. The held-out interval starts at 484 min.

Figure 5 compares the observed amplitude and phase with the full-record fit and the forecast from the training-only model. The lower panel shows amplitude residuals from both the descriptive full-record fit and the held-out forecast, making systematic temporal departures visible.

For the idealized fitted model, the rotating component changes sign after half of a slow period. Equation (13) therefore recovers the constant complex baseline exactly. This is an algebraic identity of Equation (8), not an additional verification experiment, and it does not apply to the average of two nonnegative amplitudes.

(13) $$C=\frac{\hat{z}(t)+\hat{z}(t+T/2)}{2}$$

The baseline magnitude exceeds the slow-component magnitude within the analyzed Type-A record. However, the held-out amplitude RMSE is 4.192 μm and R^2^ is −0.782, so the fixed-period model does not provide reliable extrapolation over this later interval. The data demonstrate an observed fluctuation, rather than a quantified operational safety margin or a proven absence of instability.

### 3.2. Analysis of Type-B Unit Data

Table 5 lists the Type-B 8H record, with the channel assignment established as described in Section 2.1. Its own first observation defines *t* = 0; the analysis does not require an assumed absolute acquisition date. The 34 original amplitude and integer-degree phase observations are retained unchanged.

The same complex-response fitting procedure gives an amplitude MSE of 1.473 μm^2^, RMSE of 1.214 μm, Pearson r of 0.951, and R^2^ of 0.883 for the full Type-B record. The complex RMSE is 2.089 μm, and the phase RMSE is 1.18°. Table 6 reports the refitted parameters and conditional intervals.

The amplitudes calculated from the refitted model are compared with the measured values in Table 7. The maximum absolute relative deviation is 3.28%. This lower percentage than for Type A reflects both the smaller absolute residuals and the higher measured amplitude; it is not evidence that a common physical mechanism was independently verified.

Using the fitted Type-B coefficients, Equation (14) gives the complex 1X response and its nonnegative amplitude. The time argument is in minutes, and the exponential argument is in radians.

(14) $$\hat{z_{B}}(t)=(-34.472554-74.448298i)+(5.290534-3.257259i)e^{i0.01721320625t},\hat{A_{B}}(t)=|\hat{z_{B}}(t)|$$

**Figure 6:**
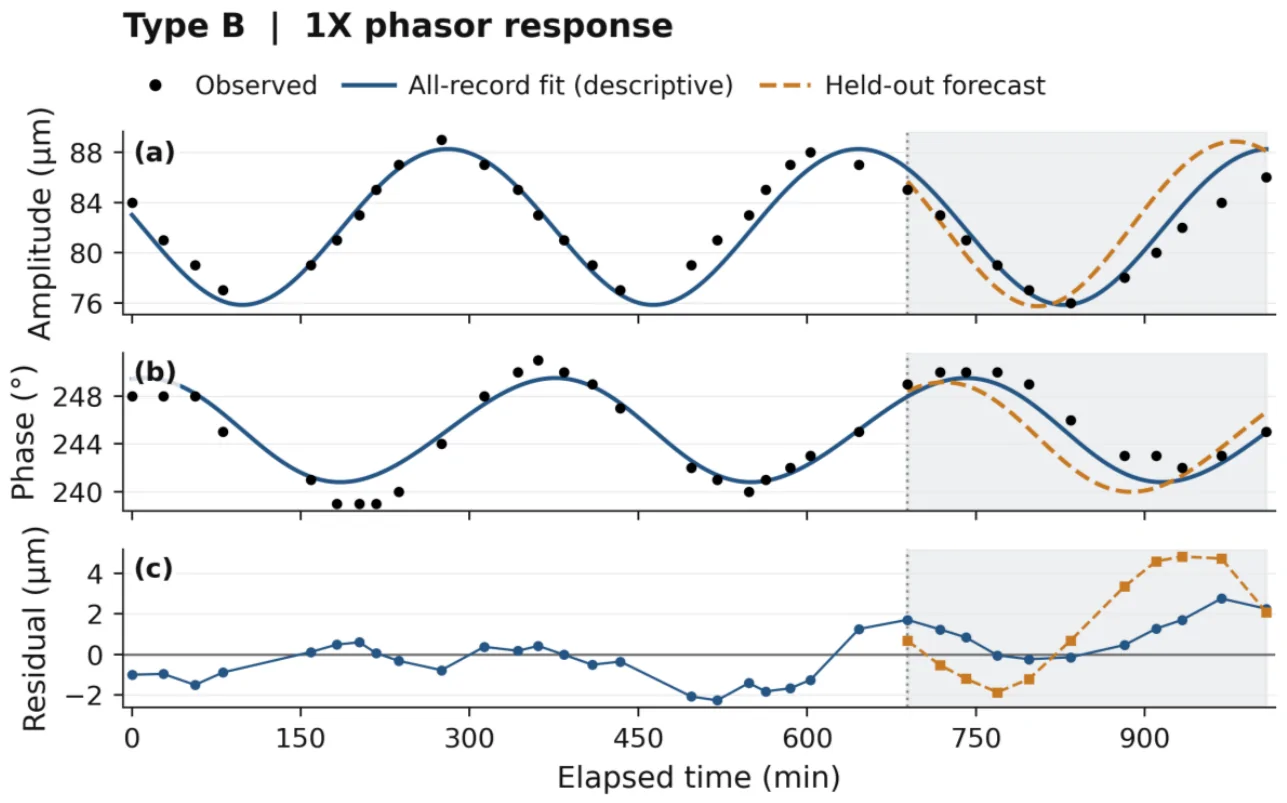
Type-B 8H amplitude, phase, and amplitude residuals. (**a**) Measured 1X amplitude versus elapsed time. Black markers are observations, blue curves are descriptive full-record fits, and orange dashed curves in the shaded interval are forecasts from the first 23 observations only; (**b**) Measured 1X phase versus elapsed time. The phase uses the recorded convention; (**c**) Amplitude residuals, calculated as predicted minus observed amplitude. Blue markers denote residuals of the full-record fit, and orange markers denote residuals of the held-out forecast. The held-out interval starts at 689 min.

The Type-B baseline magnitude is 82.04 μm, the rotating-component magnitude is 6.21 μm, and the fitted period is 365.02 min. At *t* = 0, Equation (14) gives $\hat{z}$ = −29.182020 − 77.705557i μm and $\hat{A}$ = 83.004 μm, consistent with Table 7. Its period is of the same order as the Type-A value but does not identify a shared thermal time constant. Figure 6 shows the full-record fit, training-only forecast, and amplitude residuals.

The Type-B data support a decomposition of the 8H response. Complete synchronous 6H and 7H amplitude–phase histories for the same window are not available for separate fits, so no multi-bearing comparison of fitted baseline magnitudes is inferred.

### 3.3. Comparison of the Type-A and Type-B Units

The full-record baseline magnitudes exceed the rotating-component magnitudes in both cases, but this observation concerns response vectors rather than independently identified mass unbalance. Type A has a smaller baseline and a larger relative fluctuation. Its amplitude residuals range from −3.50 to 3.08 μm, with a median of 0.06 μm and first and third quartiles of −1.31 and 0.54 μm; the corresponding Type-B values are −2.26 to 2.75 μm, −0.03 μm, and −0.94 and 0.57 μm. Correlations between residuals at successive recorded times are 0.768 and 0.805. These are ordered-record correlations rather than equal-time-lag autocorrelations because the intervals are irregular. The serial pattern indicates an unresolved temporal structure, which may include baseline drift, changing slow-component shape, or operating-condition changes; the present records do not distinguish these explanations.

Table 8 separates descriptive fit quality from temporal prediction and includes the simpler periodic amplitude model. For Type B, that baseline has a slightly lower full-record amplitude RMSE, whereas the complex model gives only a small hold-out improvement for both cases. No statistically significant superiority is claimed. The purpose of complex fitting is to preserve the phase information needed for response-vector interpretation, not to guarantee improved amplitude prediction.

The conditional 95% period intervals are 325.73–355.74 min for Type A and 356.85–373.95 min for Type B. Time-window sensitivity is larger than sensitivity to alternate-record thinning, particularly for Type A (Table 9). Ten initial periods converge to the same numerical solution within 0.001 min in each full-record fit, but numerical convergence does not remove temporal nonstationarity. The change in the Type-A period between the first and last 80% windows, from 328.88 to 366.11 min, cautions against treating one full-record period as a fixed machine property.

### 3.4. Effect of Reporting Precision

Under the stated rounding scenario, the single-observation combined quadrature contributions range from 0.301 to 0.344 μm for Type A and from 0.480 to 0.533 μm for Type B. These contributions are smaller than the full-record complex RMSE values of 3.438 and 2.089 μm. Thus, the printed integer precision alone does not explain the observed fit residuals under this assumption. Table 10 reports the propagated parameter ranges; these preserve the distinction between sensitivity to reporting precision and the broader variability captured by residual and time-window analyses.

Ranges are the 2.5th–97.5th percentiles of 2000 refits with independent ±0.5 μm and ±0.5° uniform perturbations. They describe the specified rounding scenario, not total measurement uncertainty or replacement intervals for Table 3 and Table 6. The larger variation between time windows in Table 9 remains present. The full-record P_2_/P_1_ ratios are 0.290 and 0.076 for Types A and B; these ratios remain invariant under a common, constant amplitude conversion within each record. Refitting all 59 observations after time-origin shifts of 137 and −251 min and constant phase shifts of 37° and −123° changes *P*_1_, *P*_2_ and *T* by less than 2 × 10^−9^ in relative terms. Gains of 0.5 and 2 give the expected proportional response scaling. These numerical checks leave the hold-out conclusions unchanged and do not recover alignment with thermal or load events.

### 3.5. Supporting Field Observations

A synchronous snapshot at 08:02:06 on 26 January 2018, within the Type-A analysis window, reports 5H = 11 μm ∠218°, 6H = 30 μm ∠280°, 7H = 22 μm ∠216°, and 8H = 7 μm ∠57°. These different amplitudes and phases show that the simultaneous shaft response varies across bearing locations. A single snapshot does not determine the slow component at each point or identify an inter-bearing transfer coefficient.

**Figure 7:**
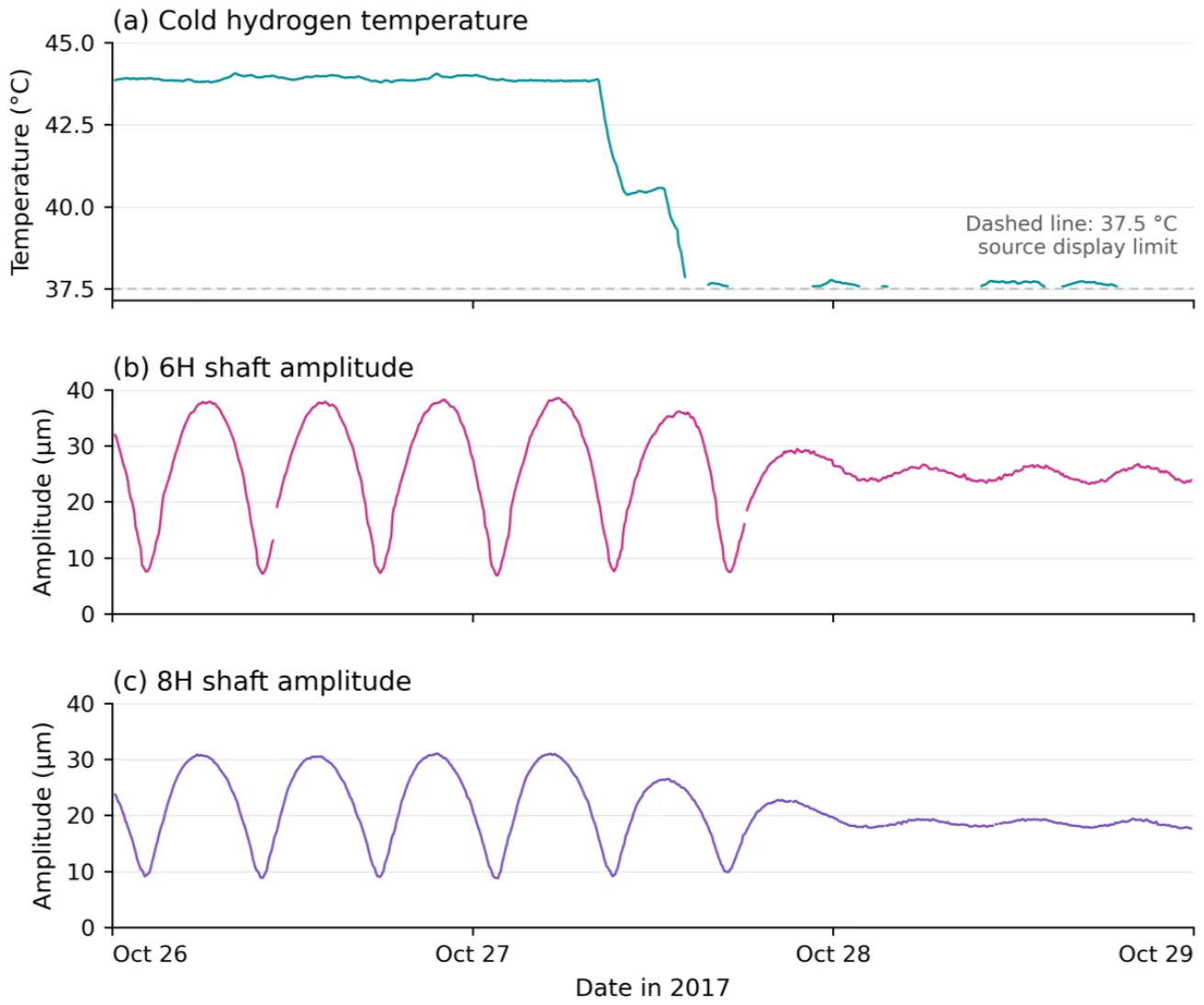
Archived cold-hydrogen-temperature and shaft-amplitude trends, 26–29 October 2017. (**a**) Cold hydrogen temperature; (**b**) 6H shaft amplitude; (**c**) 8H shaft amplitude. The visible curves were digitized from the archived monitoring plot using its labeled axis ticks. Gaps retain obscured pixels and temperature segments affected by the lower display boundary. The dashed 37.5 °C line marks that boundary, not a measured constant temperature. The amplitude labels do not establish 1X filtering; these trends were not fitted.

The archived cooling test provides a further observation of thermal sensitivity. Figure 7 shows the visible cold-hydrogen-temperature and 6H/8H shaft-amplitude trends from 26 to 29 October 2017. The temperature decreases from approximately 44 °C toward the lower display boundary, while the later portions of both amplitude traces show smaller slow excursions. The curves were digitized using the labeled time and ordinate ticks, retaining gaps where the source trace is obscured or clipped. The archived amplitude labels do not specify 1X filtering, so these trends are used as qualitative supporting observations rather than additional phasor-fit data or a controlled estimate of a temperature effect.

Following seal-ring and seat remachining, the archived monitoring window from 10 to 11 February 2018 shows no obvious recurrence of the earlier long-period excursions. The same window includes a reduction in unit load, so this is not a comparison at identical operating conditions. The inspection and subsequent operating records support attention to the seal assembly but do not isolate seal-ring mobility from load, cooling, and other concurrent changes.

A separate balancing record dated 7 February 2019 reports a 2.15 kg correction at 80° on the exciter side and the amplitudes in Table 11. Three channels decrease, whereas 7V increases from 11 to 17 μm. This mixed response demonstrates why a balancing assessment must examine all monitored directions. The record is independent of the two fitted time series; without matched complex influence coefficients, it does not validate a correction weight inferred from the Powell decomposition.

Change is the post-correction value minus the pre-correction value. The archived table does not provide phase histories or establish that these amplitudes use the same 1X extraction settings as Table 2 and Table 5.

## 4. Discussion

### 4.1. Physical Interpretation

Under a quasi-steady linear interpretation with a fixed normalized bow shape and support state, write *D_s_ = H_s_(Ω)q* for the fitted slow response coefficient (μm) at probe s. Here, q is the complex coefficient of an assumed slowly rotating bow (μm); dimensionless *H_s_* includes the displacement convention. *Ω* = 2*π* × 25 rad/s is the fast mechanical frequency; fitted *ω* (rad/min) governs slow modulation. For any positive dimensionless a, *(aH_s_, q/a)* gives the same *D_s_*. Thus, without a calibrated transfer, *P*_2_ *= |D_s_|* identifies a response magnitude but cannot uniquely recover the physical bow amplitude.

A quantitative consistency check follows directly from Equation (9): when *P*_1_ > *P*_2_, the ideal full-cycle amplitude range is *[P*_1_ *− P*_2_*, P*_1_ *+ P*_2_*]*. This gives 19.85–36.07 μm for Type A and 75.83–88.25 μm for Type B, compared with observed ranges of 17–37 and 76–89 μm. The corresponding ideal phase excursions, *2* arcsin*(P*_2_*/P*_1_*)*, are 33.71° and 8.69°, whereas the recorded excursions are 55° and 12°. Type B follows the amplitude envelope more closely; the larger phase excursion, especially in Type A, exposes departures from the circular path. This is an internal check of the retained records and fitted response path, not independent validation.

A related Arabelle shafting study found that modeled thermal excitation at the generator seals produced much larger responses at the generator bearings than at adjacent turbine bearings and did not reproduce the observed fluctuation of the full shaft train [13]. This limits any claim that local seal heating alone explains every measuring point. The present single-channel records support a response decomposition, while the spatial origin of the excitation remains a separate identification problem.

Several thermal mechanisms can produce a slowly turning 1X vector. Controlled heating experiments reproduce stable, divergent and limit-cycle spirals [21], while bearing experiments relate circumferential journal-temperature asymmetry to shaft motion and the hot-spot/high-spot phase relation [17,31]. Frictional rubbing can also generate thermal bow and a similar evolving synchronous response [32]. The studies leading to the above conclusions establish physical possibilities and discriminating measurements; they do not identify the heat source in the present units. Stationary pad-metal and coolant temperatures cannot substitute for the rotating journal-temperature field.

For the present cases, seal contact marks, cooling-associated changes and the post-maintenance monitoring window are retained as historical observations. They are compatible with seal-related thermal effects, but concurrent changes in load and cooling prevent a causal comparison. Seal-oil viscous heating, rubbing-induced heating and support-bearing thermal effects therefore remain competing explanations. Published oil-temperature studies also show that changes in viscosity act together with heat removal, clearance and support properties [17,18,19,20,24,25]. A change in the measured vibration cannot be assigned to one of these factors from the retained records.

The fitted baseline C and slow component describe displacement responses. With fixed transfer properties, fixed unbalance produces a constant phasor; changing support properties can modulate that response even if the unbalance itself is unchanged. The decomposition therefore does not separate two uniquely identified fault sources. The fitted periods correspond to modulation frequencies of 4.90 × 10^−5^ and 4.57 × 10^−5^ Hz, distinct from the 25 Hz shaft frequency. These describe the slow evolution of the retained 1X response. The retained 1X trends do not establish whether other spectral components coexist, and the residuals show departures from the assumed circular path.

### 4.2. Engineering Implications

A practical use of the decomposition begins with verifying the vibration channel and operating context, then examining whether the baseline, slow component, and residuals remain consistent across comparable time windows. Table 12 gives diagnostic rules for selecting follow-up measurements and engineering review. The increase in 7V after the separate balancing correction in Table 11 illustrates the need to verify the response across all available directions. Plant-protection actions take precedence at every stage. The cases do not establish universal numerical thresholds for *P*_1_, *P*_2_, their ratio, or seal-oil temperature. Alarm and trip decisions remain governed by the approved plant settings for the correct channel and amplitude convention; broad-band criteria [11] must not be applied directly to the fitted 1X components.

### 4.3. Scope and Limitations of the Retrospective Analysis

The analysis is limited to two short, unevenly spaced trend records and a fixed circular-path approximation. Residual structure, window sensitivity and weak Type-A hold-out performance limit prediction. The measurement scope is fixed by the retained materials described in the Data Availability Statement. Constant-reference invariance and rounding sensitivity do not establish complete measurement uncertainty or alignment with process events. The surviving model material consists of design tables and plotted summaries; the original run configuration, including the thermal-input station and output amplitude convention, is unavailable. These materials provide historical context and do not enter the 59-point phasor fit. The conditional film estimates and historical observations support discussion of possible mechanisms, while the quantitative conclusions remain limited to the recorded responses and their statistical representation.

## 5. Conclusions

This retrospective study represents slowly varying 1X vibration in two nuclear steam turbine generator records with a consistent complex response-vector model. Joint fitting of the reconstructed real and imaginary parts separates a constant response baseline from a rotating slow component. These quantities describe displacement responses and do not identify mass unbalance or thermal bow independently. The linear response framework, conditional film estimate, seal inspections and archived cooling trends provide physical context for competing explanations. The retained evidence does not establish restricted seal-ring floating as the unique source or validate a complete thermal causal chain.

For the Type-A and Type-B full-record fits, the baseline magnitudes are 27.96 and 82.04 μm, the rotating-component magnitudes are 8.11 and 6.21 μm, and the periods are 340.27 and 365.02 min. The amplitude R^2^ values are 0.918 and 0.883, and the maximum absolute relative deviations are 18.15% and 3.28%. Later observations withheld from fitting give amplitude RMSE values of 4.19 and 2.85 μm and R^2^ values of −0.782 and 0.185. These results support descriptive trend analysis but not reliable general prediction or a unique causal attribution.

Propagation of an explicit integer-rounding scenario gives central 95% period ranges of 338.99–341.53 min and 363.38–366.60 min. Their smaller widths than the residual-based intervals distinguish the limited contribution of assumed rounding from the broader residual variability and time-window sensitivity. Period and relative-component interpretation are unchanged by a constant elapsed-time origin, amplitude conversion or phase-reference conversion within a record, but these invariances do not establish predictive validity or fault attribution.

This analysis provides a transparent description of the retained vibration trends and the limits of their interpretation. The separate balancing record reduced three channel amplitudes but increased 7V, illustrating the need to consider all monitored directions. Neither a multi-point alarm ranking nor a correction weight follows from the two single-channel fits. The contribution is retrospective response characterization; the cases do not establish a validated causal diagnosis or general operating thresholds.

## Figures and Tables

**Table 1:** Reported nominal rotor properties and discretization.

Rotor	Nominal Length (m)	Density (kg/m^3^)	Young’s Modulus (GPa)	Shaft Segments	Added Masses
HIP	11.9	7857	205	61	13
LP1	12.0	7851	207	50	10
LP2	12.0	7851	207	50	10
Generator	15.2	7851	207	32	12

**Table 2:** Type-A 6H trend data and the derived imaginary phasor coordinate.

No.	Elapsed Time (min)	1X Amplitude (μm)	1X Phase (°)	1X Phase (rad)	Imaginary Coordinate Im(z) (μm)
1	0	29	309	5.3931	−22.54
2	39	22	296	5.1662	−19.77
3	64	19	282	4.9218	−18.58
4	102	23	258	4.5029	−22.50
5	127	28	254	4.4331	−26.92
6	162	33	262	4.5728	−32.68
7	183	35	267	4.6600	−34.95
8	222	37	273	4.7647	−36.95
9	271	34	282	4.9218	−33.26
10	298	31	287	5.0091	−29.65
11	314	28	290	5.0615	−26.31
12	340	23	290	5.0615	−21.61
13	359	20	287	5.0091	−19.13
14	377	18	281	4.9044	−17.67
15	390	17	274	4.7822	−16.96
16	428	21	261	4.5553	−20.74
17	458	25	259	4.5204	−24.54
18	484	29	261	4.5553	−28.64
19	511	32	263	4.5902	−31.76
20	521	33	263	4.5902	−32.75
21	541	35	266	4.6426	−34.91
22	569	36	270	4.7124	−36.00
23	634	34	278	4.8520	−33.67
24	668	30	280	4.8869	−29.54
25	693	26	277	4.8346	−25.81

**Table 3:** Full-record Type-A response parameters and conditional 95% intervals.

Parameter	Estimate	Conditional 95% Interval
*P*_1_ (μm)	27.960194	26.79 to 29.02
*θ* (rad)	−1.50599174	−1.5536 to −1.4591
*P*_2_ (μm)	8.107795	6.88 to 9.60
*ω* (rad/min)	0.01846513946	0.017662 to 0.019290
*β* (rad)	0.51472768	0.1663 to 0.8696
*T* (min)	340.272833	325.73 to 355.74

*P*_1_ and *P*_2_ are response magnitudes; *T* = 2*π*/*ω* is derived from the positive fitted slow frequency. Intervals are conditional moving-block bootstrap percentiles, not instrument uncertainty.

**Table 4:** Type-A measured and calculated 1X amplitudes from the full-record fit.

No.	Measured Amplitude *A* (μm)	Calculated Amplitude *Â* (μm)	Deviation 100(*Â* − *A*)/*A*
1	29	25.502	−12.06%
2	22	20.737	−5.74%
3	19	19.874	4.60%
4	23	22.796	−0.88%
5	28	26.334	−5.95%
6	33	31.327	−5.07%
7	35	33.693	−3.73%
8	37	35.984	−2.74%
9	34	34.378	1.11%
10	31	31.539	1.74%
11	28	29.385	4.95%
12	23	25.542	11.05%
13	20	22.883	14.41%
14	18	20.924	16.25%
15	17	20.085	18.15%
16	21	21.199	0.95%
17	25	24.962	−0.15%
18	29	28.817	−0.63%
19	32	32.390	1.22%
20	33	33.470	1.43%
21	35	35.108	0.31%
22	36	36.063	0.18%
23	34	32.063	−5.70%
24	30	27.374	−8.75%
25	26	23.716	−8.78%

**Table 5:** Type-B 8H trend data and the derived imaginary phasor coordinate.

No.	Elapsed Time t (min)	1X Amplitude (μm)	1X Phase (°)	1X Phase (rad)	Imaginary Coordinate *Im*(*z*) (μm)
1	0	84	248	4.3284	−77.88
2	28	81	248	4.3284	−75.10
3	56	79	248	4.3284	−73.25
4	81	77	245	4.2761	−69.79
5	159	79	241	4.2062	−69.09
6	182	81	239	4.1713	−69.43
7	202	83	239	4.1713	−71.14
8	217	85	239	4.1713	−72.86
9	237	87	240	4.1888	−75.34
10	275	89	244	4.2586	−79.99
11	313	87	248	4.3284	−80.66
12	343	85	250	4.3633	−79.87
13	361	83	251	4.3808	−78.48
14	384	81	250	4.3633	−76.12
15	409	79	249	4.3459	−73.75
16	434	77	247	4.3110	−70.88
17	497	79	242	4.2237	−69.75
18	520	81	241	4.2062	−70.84
19	548	83	240	4.1888	−71.88
20	563	85	241	4.2062	−74.34
21	585	87	242	4.2237	−76.82
22	603	88	243	4.2412	−78.41
23	646	87	245	4.2761	−78.85
24	689	85	249	4.3459	−79.35
25	718	83	250	4.3633	−77.99
26	741	81	250	4.3633	−76.12
27	769	79	250	4.3633	−74.24
28	797	77	249	4.3459	−71.89
29	834	76	246	4.2935	−69.43
30	882	78	243	4.2412	−69.50
31	910	80	243	4.2412	−71.28
32	933	82	242	4.2237	−72.40
33	968	84	243	4.2412	−74.84
34	1008	86	245	4.2761	−77.94

**Table 6:** Full-record Type-B response parameters and conditional 95% intervals.

Parameter	Estimate	Conditional 95% Interval
*P*_1_ (μm)	82.042099	81.40 to 82.68
*θ* (rad)	−2.00444141	−2.0157 to −1.9926
*P*_2_ (μm)	6.212848	5.37 to 7.06
*ω* (rad/min)	0.01721320625	0.016802 to 0.017607
*β* (rad)	−0.55186687	−0.7705 to −0.3119
*T* (min)	365.021206	356.85 to 373.95

*P*_1_ and *P*_2_ are response magnitudes; *T* = 2*π*/*ω* is derived from the positive fitted slow frequency. Intervals are conditional moving-block bootstrap percentiles, not instrument uncertainty.

**Table 7:** Type-B measured and calculated 1X amplitudes from the full-record fit.

No.	Measured Amplitude *A* (μm)	Calculated Amplitude *Â* (μm)	Deviation 100(*Â* − *A*)/*A*
1	84	83.004	−1.19%
2	81	80.043	−1.18%
3	79	77.502	−1.90%
4	77	76.119	−1.14%
5	79	79.123	0.16%
6	81	81.488	0.60%
7	83	83.602	0.72%
8	85	85.065	0.08%
9	87	86.687	−0.36%
10	89	88.228	−0.87%
11	87	87.377	0.43%
12	85	85.181	0.21%
13	83	83.423	0.51%
14	81	80.985	−0.02%
15	79	78.496	−0.64%
16	77	76.653	−0.45%
17	79	76.931	−2.62%
18	81	78.743	−2.79%
19	83	81.593	−1.70%
20	85	83.186	−2.13%
21	87	85.335	−1.91%
22	88	86.754	−1.42%
23	87	88.255	1.44%
24	85	86.707	2.01%
25	83	84.235	1.49%
26	81	81.840	1.04%
27	79	78.960	−0.05%
28	77	76.768	−0.30%
29	76	75.863	−0.18%
30	78	78.469	0.60%
31	80	81.271	1.59%
32	82	83.700	2.07%
33	84	86.753	3.28%
34	86	88.249	2.61%

**Table 8:** Amplitude fit and temporal hold-out comparison.

Case and Model	Full RMSE (μm)	Full R^2^	Hold-Out RMSE (μm)	Hold-Out R^2^
Type A complex	1.726	0.918	4.192	−0.782
Type A scalar periodic	1.758	0.915	4.584	−1.131
Type B complex	1.214	0.883	2.855	0.185
Type B scalar periodic	1.093	0.905	2.907	0.155

All full-record estimates are descriptive refits. Hold-out estimates use the first 17/23 observations for training and the final 8/11 for scoring in Types A/B. R^2^ uses the observed mean of the corresponding evaluation subset. No observations are randomly shuffled.

**Table 9:** Sensitivity of fitted slow period to the retained observations.

Retained Set	A n; Interval (min)	A Period (min)	B n; Interval (min)	B Period (min)
First ceil(0.8n) rows	20; 0–521	328.88	28; 0–797	357.18
Last ceil(0.8n) rows	20; 162–693	366.11	28; 202–1008	365.99
Odd-numbered rows	13; 0–693	339.14	17; 0–968	364.08
Even-numbered rows	12; 39–668	341.93	17; 28–1008	366.08

The original elapsed times are used after thinning. These tests assess sensitivity to sparse trend-record selection, not the waveform sampling frequency.

**Table 10:** Conditional parameter ranges from the reporting-precision scenario.

Parameter	Type A Estimate	Type A Central 95% Range	Type B Estimate	Type B Central 95% Range
*P*_1_ (μm)	27.960	27.851–28.070	82.042	81.941–82.139
*P*_2_ (μm)	8.108	8.025–8.196	6.213	6.094–6.330
*T* (min)	340.27	338.99–341.53	365.02	363.38–366.60

**Table 11:** Shaft amplitudes before and after the separate exciter-side balancing correction.

Channel	Before Correction (μm, Peak-to-Peak)	After Correction (μm, Peak-to-Peak)	Change (μm)
7H	15	13	−2
7V	11	17	+6
8H	58	26	−32
8V	65	21	−44

**Table 12:** Diagnostic sequence for using the response decomposition.

Step	Evidence to Examine	Engineering Action
At any stage: Operating limits	Measured protection-channel vibration or temperature reaches its approved action threshold.	Follow the existing plant action procedure; do not derive new alarm limits or oil-temperature setpoints from this fit.
1 Verify the record	Confirm channel identity, calibration, phase reference, amplitude convention, and matched speed/load history.	Resolve incompatible records before interpreting fitted phases or comparing with plant limits.
2 Evaluate the baseline	A persistent baseline across comparable windows, supported by repeatable phase and balancing-response records.	Refer for a balancing assessment using measured influence coefficients; *P*_1_ alone does not specify a correction mass.
3 Evaluate the slow component	Repeated slow variation correlated with synchronized seal-oil, metal-temperature, or clearance trends.	Review seal-ring and lubrication condition within approved procedures; consider targeted measurements or inspection.
4 Examine residuals and drift	Structured residuals, changing period, or loss of temporal hold-out performance.	Check operating changes, probe integrity, rubbing, and support variation before assigning a thermal cause.

## Data Availability

Table 2 and Table 5 contain the amplitude, phase, and elapsed-time observations used in the reanalysis. Table 1 and Appendix A report nominal rotor properties, support-bearing design inputs and outputs, per-device isolator properties and archived critical-speed comparisons. Appendix B reports conditional relative responses and digitization bounds. Figure 7 and Table 11 present auxiliary archived field observations. The original vibration waveforms, instrument calibration records and native rotor-model project are no longer recoverable, and no complete synchronized process history survives for the fitted records. Direct measurements of operating seal-ring motion, film thickness, circumferential journal temperature and thermal bow were not provided in the study record. The reanalysis is therefore confined to the retained tabulations and figures identified above.

## Appendix A. Archived Support-Bearing and Foundation Data

Table A1 reproduces archived outputs of the separate three-pad support-bearing calculation at 1500 r/min and a supply temperature of 45 °C. The source matrices are expressed in N/mm and N·s/mm; multiplication by 1000 converts them to SI units. The tabulated values use the scale factors in the caption. Equal cross-coupled entries are combined into one column, as in the solver export.

**Table A1:** Support-bearing coefficients (*K*: 10^9^ N/m; *C*: 10^7^ N·s/m).

Bearing	Load (kN)	*Kxx*	*Kxy* = *Kyx*	*Kyy*	*Cxx*	*Cxy* = *Cyx*	*Cyy*
1	491.23	1.899	0.9534	3.167	1.304	0.7288	2.426
2	651.95	2.714	1.451	4.639	1.71	0.9578	3.099
3	820.17	3.096	1.858	5.77	1.681	0.946	2.991
4	872.18	3.378	2.043	6.326	1.798	1.013	3.196
5	868.52	3.357	2.03	6.286	1.789	1.009	3.181
6	810.52	3.045	1.825	5.67	1.659	0.9338	2.954
7	1221.70	4.563	3.037	9.261	2.07	1.375	4.212
8	1191.40	4.4	2.922	8.916	2.008	1.336	4.093

The eight stiffness and damping matrices in Table A1 are symmetric and positive-definite. For any nonzero real transverse direction v, define *ρ_b_(v) = Ω (v^T^C_b_v)/(v^T^K_b_v)* at *Ω* = 157.08 rad/s, where *K* and *C* denote stiffness and damping matrices, and b indexes the bearing. The extrema follow from the corresponding generalized symmetric eigenproblem; the range over all eight bearings and directions is 0.712–1.203. Thus, the local support damping term is comparable to the stiffness term at 1X. This local comparison concerns the magnitudes of the archived support coefficients; it is not a rotor modal-damping ratio or stability estimate.

Table A2 juxtaposes the archived calculated and measured support-bearing oil-temperature rises. The tabulated series have an unweighted RMSE of 0.32 °C and a mean absolute percentage difference of 4.21%. These are descriptive differences between the reported values. The surviving table does not establish matching sensor locations and temperature-rise references, so it is not used to validate a thermal-feedback model or calibrate the phasor decomposition.

**Table A2:** Reported calculated and measured support-bearing oil-temperature rise.

Bearing	1	2	3	4	5	6	7	8
Calculated (°C)	6.4	7.0	7.1	7.3	7.3	7.1	6.6	6.6
Measured (°C)	5.9	7.2	6.9	6.9	7.5	7.6	6.5	6.8

Table A3 collects an archived critical-speed calculation and field ranges reported for startup/shutdown on 12 October 2021. Its spring-foundation model uses the mean direct support stiffness *(K_xx_ + K_yy_)/*2. The additional case retains the archive’s 50% spring-stiffness label. This table is a descriptive comparison of the archived values.

**Table A3:** Archived critical-speed and foundation-stiffness comparison (r/min).

Rotor/Mode	Without Spring Foundation	Original Spring Stiffness	50% Spring Stiffness	Reported Field Range
HIP first	1070	1022	988	967–1053
LP first (out-of-phase)	1160	1134	1015	1060–1165
LP first (in-phase)	1177	1157	1021	1060–1165
Generator first	975	955	920	916–945
Generator second	2863	2555	Not reported	Not reported

With the original spring stiffness, three of the four first-critical-speed predictions lie within the reported ranges; the generator value is 10 r/min, or 1.06%, above its range’s upper bound. The LP entries describe out-of-phase and in-phase bending of the two LP rotors; the no-foundation predictions retain their reported order, and the shared field range is not mode-resolved. These ranges are not confidence intervals. In the archived 50% stiffness case, the four first critical speeds decrease by 3.33%, 10.49%, 11.75% and 3.66%, respectively. These finite changes summarize the reported sensitivity to foundation stiffness. The second-critical-speed entries are calculated archival values only.

Table A4 and Table A5 report the archived support-bearing design inputs and spring-isolator inventory. The bearing dimensions and installation gaps follow the tabulated design specification and describe the support bearings rather than the generator seal rings.

**Table A4:** Archived support-bearing geometry and installation gaps.

Parameter	Bearings 1–2	Bearings 3–6	Bearings 7–8
Journal diameter (mm)	630	750	800
Support-pad width/arc (mm/°)	560/107	525/117	510/117
Side-pad width/arc (mm/°)	380/43	450/43	450/43
Top-pad width/arc (mm/°)	380/53	450/50	450/50
Installation gap S (mm)	0.70–0.75	0.86–0.91	0.91–0.96
Installation gap V (mm)	0.10–0.20	0.12–0.22	0.14–0.24
Reported mean (S + V)/2 (mm)	0.44	0.53	0.57
Supply-oil temperature (°C)	45	45	45

S and V retain the archived installation-gap definitions; their tabulated mean (S + V)/2 is a nominal radial-clearance input. These values do not measure the running minimum film thickness.

**Table A5:** Archived spring-isolator inventory and per-device properties.

Model	Count	Height (mm)	Vertical Stiffness (kN/mm)	Horizontal Stiffness (kN/mm)	Mass (kg)
TK-950.1612.32	6	570	30	16	452
TK-1200.2207.32	40	570	38	22	698
TK-1300.2216.32	18	570	41	22	714
TVEK-950.1612.32	12	570	30	16	787

Stiffnesses are per device (1 kN/mm = 10^6^ N/m). The 76-device inventory does not specify device locations, the foundation’s condensed matrices or its complete connections to the eight bearing nodes.

## Appendix B. Digitized Relative Responses from Archived Plots

To retain spatial amplitude information, Table A6 separately digitizes the lower-resolution four-panel archive montage and uses *R_s,p_ = (a_s,p_(*1500*))/(a*_166,1_*(*1500*))*, where s is output station and p is probe. This differs from Figure 2, which normalizes each probe by its own operating-speed amplitude. The ratios assume a common amplitude display unit and convention across the archived panels. For the stated common convention, a common unit conversion and positive scaling of the same fixed input cancel in the ratio. This table describes the relative source-plot amplitudes under that assumption.

**Table A6:** Conditional relative responses and digitization bounds.

Bearing	Output Station	Probe 1 at 5° Ratio [Bounds]	Probe 2 at 95° Ratio [Bounds]
5	117	0.60 [0.34, 1.02]	0.29 [0.13, 0.62]
6	157	0.94 [0.58, 1.54]	0.50 [0.28, 0.88]
7	166	1.00 (reference)	0.88 [0.41, 1.84]
8	191	1.01 [0.63, 1.62]	0.68 [0.41, 1.13]

Bounds allow independent ±4-pixel changes in both source-image coordinates, including numerator and reference readings; the horizontal range is approximately ±21 r/min. Limits are rounded outwards and are deterministic sensitivity bounds. The reference divided by itself is exactly one, and the overlapping bounds do not establish a uniquely largest response at bearing No. 7. The accompanying thermal-response peak summaries occur at 930–1020 r/min and are separate from these reference-speed readings. Their meter labels do not define the original solver amplitude convention.
